# PEER-LED DIET AND EXERCISE INTERVENTION FOR OLDER VETERANS WITH DYSMOBILITY

**DOI:** 10.1093/geroni/igae098.3489

**Published:** 2024-12-31

**Authors:** Ben Friedman, Monica Serra, Rhianna Patel, Elizabeth Dennis, Lisa Kilpela, Odessa Addison

**Affiliations:** University of Maryland Baltimore, Baltimore, Maryland, United States; The University of Texas Health San Antonio, San Antonio, Texas, United States; VA Medical Center, Baltimore, Maryland, United States; University of Maryland School of Medicine, Baltimore, Maryland, United States; The University of Texas Health San Antonio, San Antonio, Texas, United States; University of Maryland School of Medicine, Baltimore, Maryland, United States

## Abstract

**Purpose:**

Older adults with mobility impairments have increased falls, higher hospitalization rates, and elevated healthcare costs. Veterans face higher obesity rates and poorer health outcomes. This study evaluated the adherence and preliminary efficacy of a peer-led diet and exercise intervention targeting older Veteran with dysmobility in two urban areas with a high proportion of racial and ethnic minorities.

**Methods:**

Twelve older Veterans (78.9 ±6.4 years) from San Antonio, TX, and Baltimore, MD, participated in a 12-week peer-led intervention. The groups met twice a week for one hour and focused on behavioral modification, nutrition, and whole-body aerobic and band-resisted exercises. Baseline and post-intervention assessments included the 6 Minute Walk Test (6MWT), Timed Up and Go (TUG), grip strength,10-Meter Walk Test (10mWT), Four Square Step Test (4SST), and Short Physical Performance Battery (SPPB). Descriptive statistics were computed for each outcome and paired sample t-tests assessed within-subject changes. Adherence was measured by session attendance and completion rate.

**Results:**

Significant improvements were observed in the 10mWT (p < 0.05). While not statistically significant, most participants demonstrated improvements in the TUG (5/9), 6MWT (6/9), and 4SST (7/9). For those that improved on the SPPB (4/9), half were no longer below the cut-off score of ≤ 10. Session attendance was 60.96% (TX) and 76.47% (MD). Study completion rate was 83.33% (1 drop out per site). Implications: This pilot study supports the need for further investigation to explore the long-term effects of a peer-led diet and exercise intervention addressing multiple health domains among older Veterans.

